# Gammaherpesviral Tegument Proteins, PML-Nuclear Bodies and the Ubiquitin-Proteasome System

**DOI:** 10.3390/v9100308

**Published:** 2017-10-21

**Authors:** Florian Full, Alexander S. Hahn, Anna K. Großkopf, Armin Ensser

**Affiliations:** 1 Institute for Clinical and Molecular Virology, University Hospital Erlangen, Friedrich Alexander University Erlangen-Nuremberg, 91054 Erlangen, Germany; Florian.Full@uk-erlangen.de; 2 Nachwuchsgruppe Herpesviren, Deutsches Primatenzentrum—Leibniz-Institut für Primatenforschung, 37077 Göttingen, Germany; AHahn@dpz.eu (A.S.H.); AGrosskopf@dpz.eu (A.K.G.)

**Keywords:** Gammaherpesvirus, Epstein-Barr virus, Kaposi’s sarcoma-associated herpesvirus, KSHV, EBV, viral FGARAT, PML nuclear bodies, ubiquitin, proteasome, deubiquitinating enzyme

## Abstract

Gammaherpesviruses like Epstein-Barr virus (EBV) and Kaposi’s sarcoma-associated herpesvirus (KSHV) subvert the ubiquitin proteasome system for their own benefit in order to facilitate viral gene expression and replication. In particular, viral tegument proteins that share sequence homology to the formylglycineamide ribonucleotide amidotransferase (FGARAT, or PFAS), an enzyme in the cellular purine biosynthesis, are important for disrupting the intrinsic antiviral response associated with Promyelocytic Leukemia (PML) protein-associated nuclear bodies (PML-NBs) by proteasome-dependent and independent mechanisms. In addition, all herpesviruses encode for a potent ubiquitin protease that can efficiently remove ubiquitin chains from proteins and thereby interfere with several different cellular pathways. In this review, we discuss mechanisms and functional consequences of virus-induced ubiquitination and deubiquitination for early events in gammaherpesviral infection.

## 1. Gammaherpesviruses

*Gammaherpesvirinae* (gammaherpesviruses) is a subfamily of viruses in the family of Herpesviridae (herpesviruses) that includes a number of important human and veterinary pathogens. Gammaherpesviruses can be subdivided into the following four genera, with some exemplary viruses from each genus listed: (i) the lymphocryptoviruses (gamma-1) with the human pathogen *Epstein-Barr virus* (EBV) and related primate viruses; (ii) the rhadinoviruses (gamma-2) with the human pathogen *Kaposi’s sarcoma-associated herpesvirus* (KSHV); the prototypic rhadinovirus *Herpesvirus saimiri* (HVS); the *Rhesus monkey rhadinovirus* (RRV); and the *murid herpesvirus 4* (MuHV4, MHV-68). A reappraisal of taxonomy based on their biology led to a reclassification of several former gamma-2 herpesviruses into the (iii) percaviruses with *Equid herpesvirus 2* (EHV-2) and the (iv) macaviruses with *Alcelaphine gammaherpesvirus 1* (AlHV-1).

KSHV (Human Herpesvirus-8, HHV-8), and Epstein-Barr Virus (Human Herpesvirus-4, HHV-4) are the only gammaherpesviruses known to cause human disease. Above all, EBV and KSHV are unique among human herpesviruses in their ability to cause cancer in humans. EBV is associated with multiple forms of human cancer, including Burkitt’s lymphoma, nasopharyngeal carcinoma, some forms of Hodgkin’s lymphoma, and lymphoproliferative disease in immunocompromised patients, including those with AIDS [1]. KSHV is the etiologic agent of three human tumor-diseases: Kaposi’s sarcoma (KS), a skin tumor of endothelial origin, and two lymphoproliferative B-cell malignancies, the Primary Effusion Lymphoma and a plasmablastic variant of Multicentric Castleman’s Disease [2]. KS exists in three forms, classical KS, which is endemic in the Mediterranean and Middle East [3], iatrogenic KS and AIDS-associated KS, which ranks among the leading causes of death in AIDS patients [4]. Whereas the incidences of tumors caused by KSHV and EBV are moderate in western countries, both viruses are major causes of cancer and subsequent morbidity and mortality in sub-Saharan Africa [5]. In this region, KS ranks as the fourth most common cancer in the general population. Especially the interplay of KSHV with HIV, and of EBV with Malaria and HIV, contributes to cancer in sub Saharan Africa. To this end, a better understanding of gammaherpesviral carcinogenesis is important to identify new targets for antiviral therapy.

## 2. The Ubiquitin-Proteasome System

Ubiquitination is a posttranslational protein modification that is involved in multiple cellular processes in eukaryotes. Ubiquitin is a small protein of 76 amino acids that is attached to lysine residues of proteins by a cascade of enzymes. Ubiquitin first needs to be activated by E1 enzymes, is then conjugated by E2 enzymes and finally ligated by E3 ligases [6]. The specificity for the substrate protein is determined by the E3 ligase whereas the primary function of the E2 enzymes is to determine which types of polyubiquitin chains are catalyzed by the E3. In addition, ubiquitin can also be removed from proteins by a group of deubiquitinating enzymes (DUBs). Ubiquitination may result in mono- or polyubiquitylated proteins. For polyubiquitination, either the aminoterminal methionine or one of the seven internal lysine residues of ubiquitin can be modified in consecutive conjugation cycles, leading to long ubiquitin chains. The resulting ubiquitin chains can either be linear, if connected by the same type of linkage, or branched, if different types of linkages are used within one chain [7]. The different types of ubiquitin linkage determine the fate of the modified protein. Whereas K48-linked polyubiquitin chains are recognized by the proteasome and result in degradation of the substrate, K63-linked ubiquitination does not usually trigger proteasomal degradation, but plays crucial roles in signal transduction pathways [7].

The cellular ubiquitin-proteasome system represents a unique opportunity for viruses to eliminate unwanted cellular proteins. As a pre-existing fast-track system for protein degradation, it obviates the need for the virus to encode its own enzymatic activity. Only the targeting part of the system, the E3 ligase needs to be subverted. The enzymatic nature of the ubiquitin-proteasome system allows for very efficient degradation of large amounts of host proteins requiring only small amounts of viral protein, when compared to the stoichiometric amounts required for sequestration of cellular targets. Given these highly attractive features of the ubiquitin-proteasome system, it is no surprise that viruses have evolved multiple ways to make use of this system, in particular for the degradation of cellular restriction factors.

## 3. PML Nuclear Bodies

In the herpesviral virion, the viral tegument is the matrix of proteins that fills the space between the nucleocapsid and the viral envelope. It is composed of structural proteins necessary for the formation of the viral particle and of viral effector proteins. During infection, viral tegument proteins are released into the cytoplasm and also transported to the nucleus of the host cell. They facilitate viral infection and viral gene expression by subverting the host innate immune response and enhancing viral transcription and translation. Upon release of the viral genome from the capsid into the nucleus, the incoming viral genomes have been shown to associate with subnuclear structures called PML-nuclear bodies (PML-NBs, also designated Nuclear Domain 10 (ND10)). PML-NBs are named after their key structural component, the promyelocytic leukemia protein (PML or TRIM19). PML-NBs have been implicated in various biochemical nuclear functions, for example they have been described as sites of active gene expression, epicenters of posttranscriptional modification of proteins, in particular SUMOylation, and as storage centers for nuclear proteins [8]. In addition, it became evident in recent years that PML-NBs also contribute substantially to the intrinsic immunity to viral infection, in particular to infection by DNA viruses [9]. PML-NB components PML, SP100, DAXX and ATRX are bona fide restriction factors for viral infection and members of different virus families have evolved sophisticated mechanisms to bypass PML-NB-mediated immunity. Numerous reports that were published over the past 10–15 years identify PML-NBs as part of the first line of cellular defense against DNA virus infection. Notably, the expressions of PML-NB components such as PML and SP100 themselves are also induced by the interferon pathway, corroborating their crucial role in the intrinsic immunity to viral infection [10].

## 4. PML-NBs as Targets of Gamma-Herpesviral FGARATs

The disruption of PML nuclear bodies has first been observed for the alpha- and beta-herpesviruses HSV-1 and HCMV [11,12]. The HSV-1 regulatory protein ICP0 has been shown to induce the proteasomal degradation of PML either via acting as a SUMO-targeted ubiquitin ligase or via SUMO-independent ubiquitination [13,14]. Similarly, the IE1 protein of human cytomegalovirus targets PML by inhibiting its de novo SUMOylation [15]. Since SUMOylation of PML is essential for the integrity of PML-NBs, this leads to a complete loss of PML-NBs and dispersal of other PML-NB proteins throughout the nucleus.

**Figure 1:**
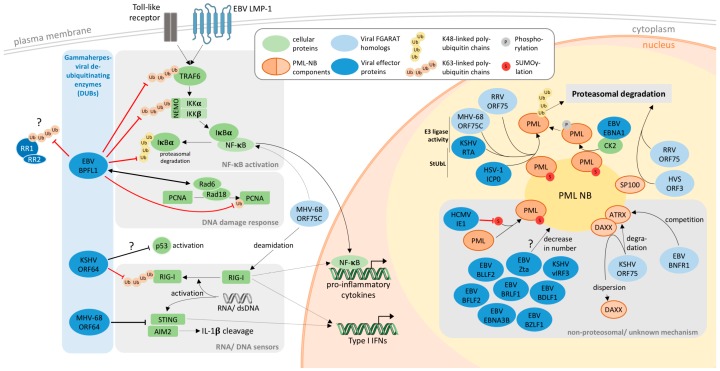
Modulation of the host ubiquitin-proteasome system and the innate immune response by gamma-herpesviral effector proteins. Gamma-herpesviral ORF64 homologs act as viral ubiquitin proteases interfering with different cellular pathways (grey boxes) resulting for example in a decreased Type I interferon response and pro-inflammatory cytokine production (left). Viral proteins, including the viral formylglycineamide ribonucleotide amidotransferase (FGARAT) homologs (light blue) disrupt the intrinsic antiviral response associated with PML-NBs by targeting different PML-NB components (right) through proteosomal and non-proteosomal/ unknown modes of action (grey box). Abbreviations: Ub, ubiquitin; S, SUMOylation; P, phosphorylation; StUbL, SUMO-targeted ubiquitin ligase; RR1, large subunit of EBV ribonucleotide reductase; RR2, small subunit of EBV ribonucleotide reductase; LMP-1, latent membrane protein 1. The Herpes simplex ICP0 and human Cytomegalovirus IE1 proteins were included for reference. Red bar-headed lines indicate removal of post-translational modifications, black bar-headed lines indicate inhibition, ? indicates unknown mechanisms; arrow-headed lines indicate interaction; solid lines indicate direct interaction, dotted lines indirect interaction; the yellow area represent PML-NB-associated functions.

The first gammaherpesvirus that has been shown to disrupt PML-NBs was EBV (The modulation of the host ubiquitin-proteasome system and the innate immune response by gamma-herpesviral effector proteins is summarized in Figure 1). The viral Z-transactivator regulatory protein Zta, which is critical for productive lytic infection, was identified for being necessary and sufficient for the disruption of PML-NBs [16,17]; however it does not mediate degradation of PML. A seminal work by the group of Lori Frappier demonstrated that overexpression of at least six EBV-encoded proteins (BDLF1, EBNA3B, BRLF1, BFLF2, BLLF2, BZLF1) can reduce the average number of PML-NBs per cell [18], underlining the importance of this structure during the viral lifecycle. Later, the EBNA1 protein was also discovered to disrupt PML bodies. EBNA1 achieves this through recruitment of cellular casein kinase 2 (CK2) to PML-NBs, increasing the phosphorylation of PML, which in turn increases ubiquitination and proteasomal degradation of PML [19,20].

Infection with murid herpesvirus 4 (also called murine herpesvirus 68 or MHV-68) also results in the dissolving of PML-NBs [21,22]. The effect of ectopic expression of several MHV-68 candidate proteins on PML-NBs was tested and ORF75C identified as responsible effector protein. ORF75C of MHV68 mediates the proteasomal degradation of PML, which results in a loss of PML nuclear bodies and results in enhanced viral gene expression and replication. Interestingly, Sewatanon et al. were able to demonstrate that ORF75C itself contains E3 ligase activity, obviating the need to recruit additional cellular factors other than UBE1 and UBE2-type molecules [23].

All gammaherpesviruses encode one to three FGARAT-homologous proteins (viral FGARAT or vFGARAT) in their genome, EBV BNRF1, KSHV ORF75, RRV ORF75, HVS ORF3 and ORF75, MHV68 ORF75A, ORF75B and ORF75C. The homology of viral teguments proteins with the cellular formylglycineamide ribonucleotide amidotransferase enzyme or phosphoribosyl formylglycinamidine synthase (FGARAT or PFAS, EC 6.3.5.3) was first recognized by Ensser et al. in the AlHV1 [24]. Over the past ten years, several studies showed that vFGARAT family members of different gammaherpesviruses modulate different PML-NB proteins (summarized in Table 1), suggesting that the PML-NB targets of the viral FGARAT protein family have diversified during evolution [25,26]. All vFGARAT proteins analyzed so far are tegument proteins and part of the virion. Thus, one obvious function of vFGARATs, besides their role as structural component of the virion, is the antagonization of PML-NB mediated intrinsic immunity [26].

For KSHV we recently demonstrated that ORF75 mediates the degradation of ATRX and redistribution of DAXX, whereas other PML-NB proteins were not affected by KSHV-ORF75 [29]. Ectopic expression of ORF75 alone is sufficient to induce the reorganization of PML-NBs; it leads to less PML-NBs but with bigger diameter and irregular shape. The degradation of ATRX by ORF75 was independent of the proteasome, since proteasome inhibition was not able to reverse the observed phenotype; the exact mechanism for degradation of ATRX still has to be resolved [29]. Unexpectedly, a mutant KSHV lacking the entire *orf75* gene completely lost the ability to replicate in cell culture in contrast to wildtype virus, and no viral gene expression could be detected in the absence of ORF75. In contrast to KSHV, the closely related HVS exclusively degrades the cellular ND10 component SP100 via the proteasome whereas other factors like PML, ATRX or DAXX remain intact [25]. The vFGARAT tegument protein ORF3 of HVS was identified as being responsible for this effect. ORF3 induced the proteasomal degradation of SP100, and a mutant HVS lacking the *orf3*-gene was no longer able to mediate SP100 degradation [25]. Infection of fibroblasts with HVS results in restriction of immediate-early gene expression, which can be alleviated by siRNA mediated knock down of PML [25]; PML is thus also a restriction factor for HVS infection. In a similar fashion, the vFGARAT of RRV, ORF75, induces degradation of both PML and SP100 upon recombinant expression, and infection with RRV leads to an almost complete loss of these ND10 components in infected cells [33]. In line with these results, CRISPR-mediated knockout of PML or SP100 did not increase infection by RRV, as opposed to knockout of other PML-NB components like DAXX, which is not targeted by RRV ORF75. In cells that did not support lytic replication of RRV, or in cells recombinantly expressing RRV ORF75, loss of PML and SP100 was prevented by inhibition of the proteasome. This demonstrates that RRV subverts this cellular degradation pathway to get rid of host restriction factors [33].

For EBV, Tsai et al. demonstrated that the vFGARAT BNRF1 binds to DAXX and outcompetes binding of DAXX to ATRX, thereby disrupting the DAXX-ATRX complex and facilitating viral gene expression [27]. By disrupting the complex, BNRF1 suppresses DAXX-ATRX-mediated Histone H3.3 loading on viral chromatin and thereby enhances viral gene expression [28,34]. This attack on components of the PML-NB also does not involve the proteasome. As mentioned above, at least one other effector protein of EBV, EBNA1, does make use of the ubiquitin-proteasome system to get rid of PML-NB components. Among the vFGARAT, ORF75C of MHV-68 is the only family member that has been attributed an intrinsic ubiquitin E3-ligase activity and directly mediates the proteasomal degradation of target proteins [23]. It remains to be determined whether the two vFGARATs of HVS and RRV that mediate proteasomal degradation of PML-NB components also contain E3 ligase activity or need to recruit additional cellular factors.

During the coevolution with their respective hosts, the viruses and the function of vFGARATs have evolved in a way that reflects the biological behavior of the different viruses. It is remarkable that vFGARATs of viruses like MHV-68 or RRV, that are capable of full lytic replication in vivo and in vitro, are degrading PML, which results in a complete disruption of ND10-NBs and thus efficient viral IE-gene expression. In contrast, viruses like KSHV, for which the default outcome of infection in most if not all cells is latency, encode for vFGARATs that are not capable of degrading PML but affect other components of ND10-NBs like SP100, DAXX or ATRX, and often in a more subtle way than plain degradation.

The absence of viral gene expression and viral replication in a KSHV orf75-null mutant virus was unexpected at first [29]. Similarly, recombinant RRV orf75-null mutant was also found to be replication deficient [33]. Nevertheless, vFGARATs are not generally essential for gammaherpesviral replication. Other gammaherpesviruses with vFGARAT deletion are able to form infectious viral particles; a BNRF1-null virus is capable of viral replication, the same holds true for an MHV68-orf75c virus, and orf3- or orf75 deleted HVS [22,25,35]. HVS encodes for two vFGARAT proteins; the *orf3* gene is located near the left end of the genome and the *orf75* gene is located at the right end of the genome. The separate deletion of orf3 or orf75 each results in a two-log decrease in viral titer, but still results in production of replication competent viral particles. HVS with a simultaneous deletion of orf3 and orf75 from its genome, however, is not replication competent (Full and Ensser, unpublished). This could be explained by structural requirements, as vFGARATs are integral components of the virion. For EBV, a BNRF1-null virus mutant is still able to replicate and to form infectious particles in the absence of the vFGARAT protein. EBV BNRF1-null virion infection is nevertheless abortive, as the viral genome is not delivered to the nucleus [35]. Thus, at least one vFGARAT gene seems to be required for viral gene expression and replication of the rhadinoviruses KSHV, RRV and HVS, whereas for replication of the lymphocryptovirus EBV its single vFGARAT gene seems to be dispensable. Whether this is a general distinction between rhadinoviruses and lymphocryptoviruses and whether differences in functional characteristics in respective vFGARATs contribute to this observation needs to be the subject of further investigation.

Recently, it was also reported that infection with EBV leads to an overduplication of centrosomes and aneuploidy [36]. Excess in the number of centrosomes per cell is a hallmark of cancer cells, can be observed as a consequence of mutations in tumor-suppressor genes like p53 or oncogenes like BRCA1, and is associated with chromosomal instability [37]. Centrosome amplification did not occur after infection with a BNRF1-null-EBV compared to wildtype-EBV [36]. Moreover, ectopic expression of BNRF1 was sufficient to induce centrosome amplification leading to aneuploidy in expressing cells, indicating a direct effect of BNRF1 on centrosome numbers and genome stability [36]. The detailed mechanism has to be elucidated, e.g., whether proteasomal degradation of a regulator of the centrosome cycle may be involved. The finding raises the question if vFGARAT proteins are potential oncogenes, because centrosome overduplication in tumors is usually induced by oncogenes. Centrosome overduplication is associated with aneuploidy, which cannot be observed in early KS tumor cells or HVS and EBV transformed lymphocytes, but centrosome overduplication is also associated with asymmetric cell divisions, reorganization of the cytoskeleton, changes in cell polarity and altered signaling, which can contribute to tumor invasion and tumorigenesis [38]. Similar to BNRF1, the KSHV homologue ORF75 is also expressed at low levels in latently infected cells and although speculative, it is quite remarkable in this context that among the human herpesviruses, only EBV and KSHV are capable of tumor formation and only EBV and KSHV encode for a vFGARAT protein. Thus, vFGARATs might at least contribute to viral tumorigenesis and further studies will be necessary to assess these questions.

In addition to vFGARATs, several other gammaherpesviral proteins have been shown to colocalize with ND10-NBs, including KSHV vIRF3 (LANA2, K10.5), which partially colocalizes with PML, and whose overexpression is associated with a redistribution of PML and reduction of PML-NB numbers [30,31]. However, the contribution of IFN-regulation by vIRFs has to be taken into consideration, e.g., preventing IFN mediated upregulation of PML or SP100 also reduces PML-NBs. Another KSHV gene, ORF K8 encodes an early lytic protein, which shares homology to the EBV BZLF1 gene. K8 is a SUMO interaction motif (SIM) dependent, SUMO-2/3-specific SUMO E3 ligase that colocalizes with PML [39,40]. K8 recruits p53 and probably sequesters it to PML bodies but has no function in dispersing PML bodies.

Reinforcing the notion that PML-NB are critical obstacles to early gene expression of the herpesviruses, the lytic switch protein, RTA, of KSHV has been found to act as E3 ligase, ubiquitinating PML and inducing its degradation [32]. Similarly to ICP0 of HSV-1, KSHV RTA acts as a SUMO-targeted ubiquitin ligase (StUbL). The protein contains several SUMO interaction motifs that can bind to SUMO2/3-modified proteins with high affinity and can mediate the proteasomal degradation of SUMOylated proteins including PML [32].

## 5. Gammaherpesviral DUBs

ORF64 is a tegument protein with homologs in all herpesviruses, KSHV ORF64 interacts with capsid proteins ORF25, ORF26 and ORF62 and also with various tegument proteins and KSHV glycoproteins [41]. It is assumed that ORF64 plays an important structural role in the virion and is critical for virion production. Deletion of herpesviral ORF64 homologs is either lethal for the virus or leads to a significant drop in virus production for all viruses tested so far [42,43]. ORF64 forms a structure in the virion that serves as docking station for other tegument proteins during tegumentation [41]. In addition, ORF64 and its homologs are potent DUBs [44,45]. The DUB activity of the ORF64 homologue of HSV-1, UL36 is restricted to K48-linked ubiquitin chains [46] and the HCMV homologue UL48 prefers K63-linked ubiquitin chains [47]. In contrast, KSHV ORF64 and the EBV homologue BPLF1 have been shown to be able to remove both K48- and K63-linked ubiquitin chains from interacting proteins [44,48], thereby interfering with a number of ubiquitin-mediated cellular processes, including protein degradation and innate immune signaling. Herpesviral DUBs are among the largest proteins encoded by herpesviruses (about 280 kDa), and their DUB activity has been located to the N-terminus. For BPLF1 it has been demonstrated that the DUB domain is released from the full-length protein by Caspase-1 cleavage [49]. Cleavage of the DUB domain from the full-length protein is even essential for DUB function of HSV-1 UL36 [46] and has also been reported for UL48 and M48 of HCMV and MCMV [50,51], which points at a conserved cleavage mechanism for herpesviruses. As part of the virion, herpesviral DUBs are delivered into the newly infected cells and can directly exert their DUB activity on target proteins. As ubiquitination, in particular non-degradative K63-linked ubiquitination, is an important regulator of the activity of many pathogen-associated pattern receptors [52], viruses can use their deubiquitinating activity to block innate immune signaling and facilitate viral replication. In this context, it was reported that KSHV ORF64 interferes with viral activation of the innate immune sensor RIG-I by removing K63-linked ubiquitin chains from RIG-I, which is essential for its activation [53]. Interestingly, it could be shown that the vFGARATs of KSHV and MHV68 also interfere with RIG-I signaling by a novel mechanism. vFGARATs that lost their enzymatic activity are capable of recruiting cellular FGARAT to RIG-I, redirecting the enzymatic activity of the cellular enzyme to deamidate RIG-I, which results in RIG-I activation and a block of IFN-signaling [54]. Moreover, MHV-68 ORF64 has been shown to interfere with STING/ AIM2 signaling [55]. This leads to a decrease in IFN-production and thereby promotes viral replication. In addition, BPLF1 prevents NF-κB activation and proinflammatory cytokine production by Toll-like receptor (TLR) signaling during productive EBV infection. BPLF1 interferes at multiple levels in the TLR signaling cascade by removing K63-linked ubiquitin from TRAF6 and NEMO, and K48-linked ubiquitin from IκBα [48]. Using a slightly different model system, Saito et al. showed that BPLF1 is also able to facilitate virus production by inhibiting LMP-1 mediated NF-κB activation through deubiquitination of TRAF6 [56].

Interestingly, BPLF1 also has a viral protein as target: it was shown that it interacts with the large subunit of the EBV ribonucleotide reductase (RR) and removes ubiquitin chains from RR, thereby decreasing RR activity in an in vitro assay [57]. The functional consequences of this interaction however are unknown and need to be addressed in further studies, especially in light of the fact that the RR of MHV-68, ORF61, colocalizes with PML and reorganizes PML into track-like structures [58]. BPLF1 is also known to interfere with cellular DNA repair pathways by deubiquitination of the cellular processivity factor PCNA, by interacting with the RAD6/RAD18 ubiquitin complex, and by promoting the nuclear translocation of DNA polymerase eta, a polymerase involved in DNA repair processes [59,60,61]. Moreover, KSHV ORF64 has been shown to interfere with p53 activation, however the detailed mechanism is not clear [62]. Aside from its role as potent DUB, BPLF1 can also act as a de-NEDDylase. BPLF1 is a potent de-NEDDylase for Cullins, cellular proteins that serve as scaffold for Ring proteins to form Cullin Ring ubiquitin ligases (CRLs) [63]. BPLF1-mediated de-Neddylation modulates CRL activity by proteasomal degradation of Cullins and stabilization of CRL substrates, affecting the cell cycle and promoting viral replication [49,63].

In summary, the small and large gammaherpesviral tegument proteins, despite being relatively divergent between viruses and even within the same virus, have evolved viral effector mechanisms that, even if not fully elucidated today, converge on a particular cellular target structure, the PML-NB. While unlike HVS, RRV, and MHV-68, KSHV and EBV tegument proteins do not directly induce proteasomal degradation of the major PML-NB components PML or SP100, they encode at least one other effector protein fulfilling exactly that function, KSHV RTA and EBV EBNA1. For the gamma-herpesviruses, proteasomal degradation of PML-NB components stands out as one of the principle modes of action of their viral effector proteins. Probably more subtle but also extremely effective, the gammaherpesviral DUBs influence a wide range of cellular signaling cascades that rely on post-translational modification of proteins with ubiquitin or ubiquitin-like modifiers.

## Figures and Tables

**Table 1:** Gammaherpesviral effector proteins that interfere with components of PML-NBs.

Virus	Protein	PML-NB Target	Mechanism	Reference
*Epstein-Barr virus*	BNRF1	ATRX, DAXX	disruption of DAXX/ATRX binding	[27,28]
*Epstein-Barr virus*	EBNA1	PML	proteasomal degradation	[20]
*Epstein-Barr virus*	BZLF1	PML	redistribution	[16,17]
*Kaposi’s sarcoma-associated herpesvirus*	ORF75	ATRX, DAXX	unknown, redistribution	[29]
*Kaposi’s sarcoma-associated herpesvirus*	vIRF3	PML	redistribution	[30,31]
*Kaposi’s sarcoma-associated herpesvirus*	RTA	PML	proteasomal degradation	[32]
*Rhesus Rhadinovirus*	ORF75	SP100, PML	proteasomal degradation	[33]
*Herpesvirus saimiri*	ORF3	SP100	proteasomal degradation	[25]
*Murine herpesvirus 68*	ORF75C	PML	proteasomal degradation	[21,22,23]
